# Novel inhibitors of the (VIBVN) NAT protein identified through pharmacophore modeling

**DOI:** 10.1038/s41598-025-85869-4

**Published:** 2025-01-23

**Authors:** Wei Wei, Xionghao Li, Ning Hou, Aowei Xie, Huicong Liang, Ting Gao, Xiaoli Jing, Liqin Li, Jiejie Hao, Ximing Xu

**Affiliations:** 1 Affiliated Huzhou Hospital, The Key Laboratory of Molecular Medicine, Zhejiang University School of Medicine, Huzhou Central Hospital, The Fifth School of Clinical Medicine of Zhejiang Chinese Medical University, Huzhou, 313000 China; 2 Key Laboratory of Marine Drugs, School of Medicine and Pharmacy, Ministry of Education, Ocean University of China, Qingdao, 266071 China; 3 Marine Biomedical Research Institute of Qingdao, Qingdao, 266071 China; 4 Network and Information Center, Qingdao Marine Science and Technology Center, Qingdao, 266237 China; 5 College of Food Science and Engineering, Ocean University of China, Qingdao, 266071 China; 6 TCM Key Laboratory Cultivation Base of Zhe jiang Province for the Development and Clinical Transformation of Immunomodulatory drugs, Huzhou Central Hospital, Huzhou, 313000 China

**Keywords:** Enzymes, Chemical biology, Computational biology and bioinformatics, Drug discovery

## Abstract

Arylamine *N*-acetyltransferases (NATs, E.C. 2.3.1.5) constitute a family of phase II drug metabolizing enzymes. These enzymes catalyze the transfer of acetyl groups from acetyl-CoA to a variety of substrates including arylamines, arylhydrazines, and N-hydroxyarylamines. By facilitating these reactions, NATs play a pivotal role in the detoxification and metabolic processing of a wide range of drugs and carcinogens. NAT in marine *V. vulnificus* plays a role in the metabolism of drugs, leading to the development of drug resistance in marine *V. vulnificus*. However, inhibitors targeted marine *V. vulnificus* NAT [(VIBVN)NAT] remain unclear. Therefore, our research aimed to identify potential hit compounds that target (VIBVN)NAT. We integrated multiple computational approaches to screen for effective inhibitors. From this process, we identified two hit compounds, AK-968-11563024 and AG-205-36710025, with IC_50_ values of 18.86 µM and 33.27 µM, respectively. Molecular dynamics simulations further elucidated the binding mechanism between (VIBVN)NAT and AK-968-11563024. Our study revealed that AK-968-11563024 forms stable interactions with PHE124, HIS167, and TRP230, which may contribute to its biological activity. Our findings provide a valuable foundation for the future development of drugs targeted therapeutics against (VIBVN)NAT.

**Supplementary Information:**

The online version contains supplementary material available at 10.1038/s41598-025-85869-4.

## Introduction

As a member of the Vibrio family, *Vibrio-vulnificus* (*V. vulnificus*) is a gram-negative bacterium pervasively found in marine. *V. vulnificus* has emerged as one of the most formidable global pathogens due to its extensive genetic diversity and high pathogenicity^1^. In the United States, this extremely harmful pathogen is responsible for approximately 90% of seafood-related fatalities and is implicated in about half of all cases in immunocompromised individuals and other vulnerable groups^2,3^. It is essential to have precise and accessible therapeutic interventions against this bacterium in the clinical setting. Previous studies have reported that *V. vulnificus* was commonly susceptible to multiple antibiotics, such as tetracyclines, cephalosporins, chloramphenicol, et al.^4,5^. Nevertheless, recent studies have shown that *V. vulnificus* has evolved to exhibit resistance against a broad spectrum of these antibiotics^6^.

Arylamine N-acetyltransferases (NATs, E.C. 2.3.1.5) are a family of phase II drug-metabolizing enzymes^6^. They are known for detoxifying a wide range of drugs and carcinogens^7^, playing a vital role in protecting *V. vulnificus* against both aromatic compounds and probiotic bacteria^8^. NATs facilitate the transfer of acetyl groups from acetyl-CoA (AcCoA) to the terminal nitrogen of hydrazine and arylamine drugs and carcinogens^9^. NATs inactivate drugs at different rates, including the antituberculosis drug isoniazid and the antihypertensive drug hydralazine^9^. As a result, there is increasing interest in the important role these enzymes play in pharmacology.

We had evaluated the effect of (VIBVN)NAT on the acetyl conversion of arylamine compounds in previous studies^10^. In this study, we predicted the three dimensional (3D) structure of (VIBVN)NAT, and through the analysis of the thermodynamic properties of water molecules to predict drug-likeness of the binding pocket. Structure-based virtual screening methods were employed to screen the Specs database. As a result, we identified two novel inhibitors targeting NAT, AK-968-11563024 (IC_50_, 18.86 µM) and AG-205-36710025 (IC_50_, 33.27 µM), respectively. Ultimately, through the molecular dynamics (MD) simulations, we found that AK-968-11563024 establishes stable interactions with PHE124, HIS167, and TRP230, which may contribute to its biological activity. In conclusion, we innovatively reported a valuable inhibitor that lays the groundwork for the development of future targeted therapeutics against (VIBVN)NAT.

## Results

**Fig. 1:**
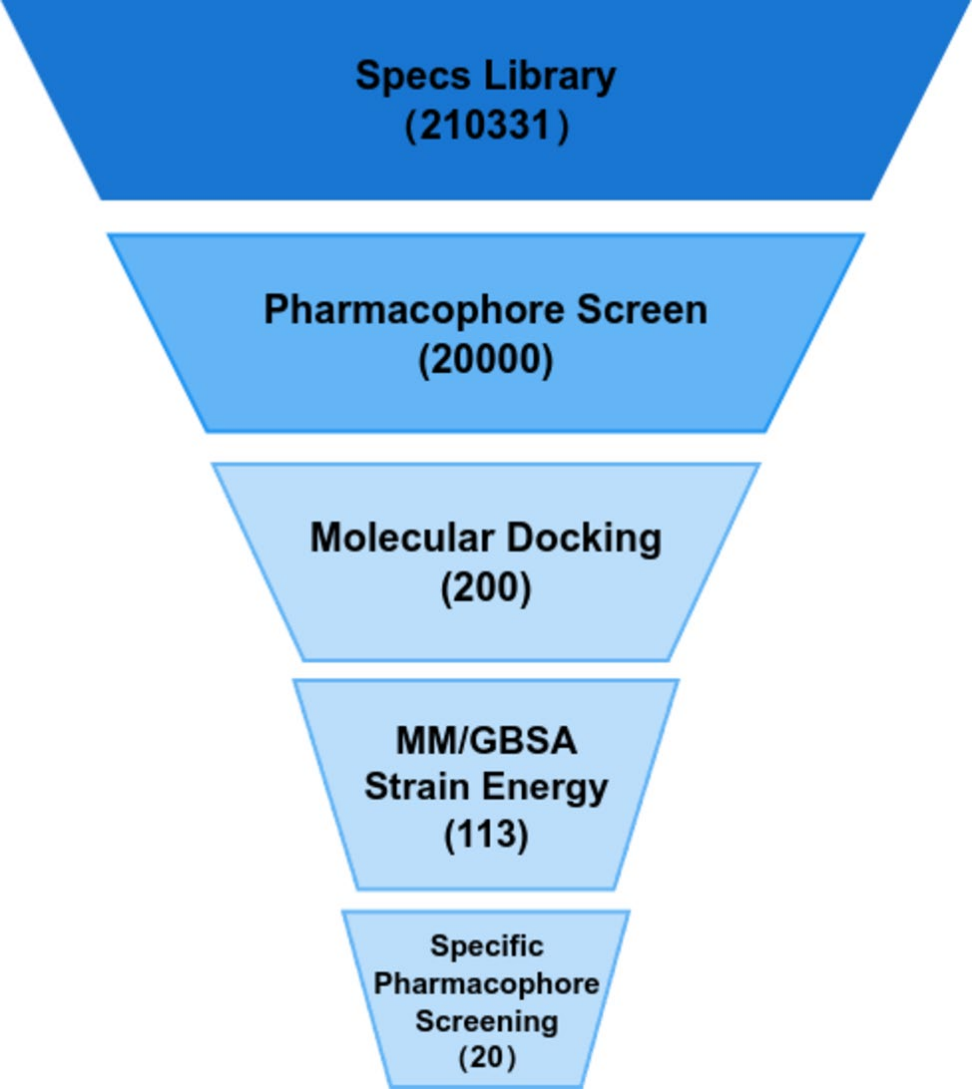
Virtual screening workflow.

The (VIBVN)NAT plays a crucial role in the detoxification of many drugs and carcinogens. Our research primarily focused on the identification of potential inhibitors (Fig. 1).

### (VIBVN)NAT structure

**Fig. 2:**
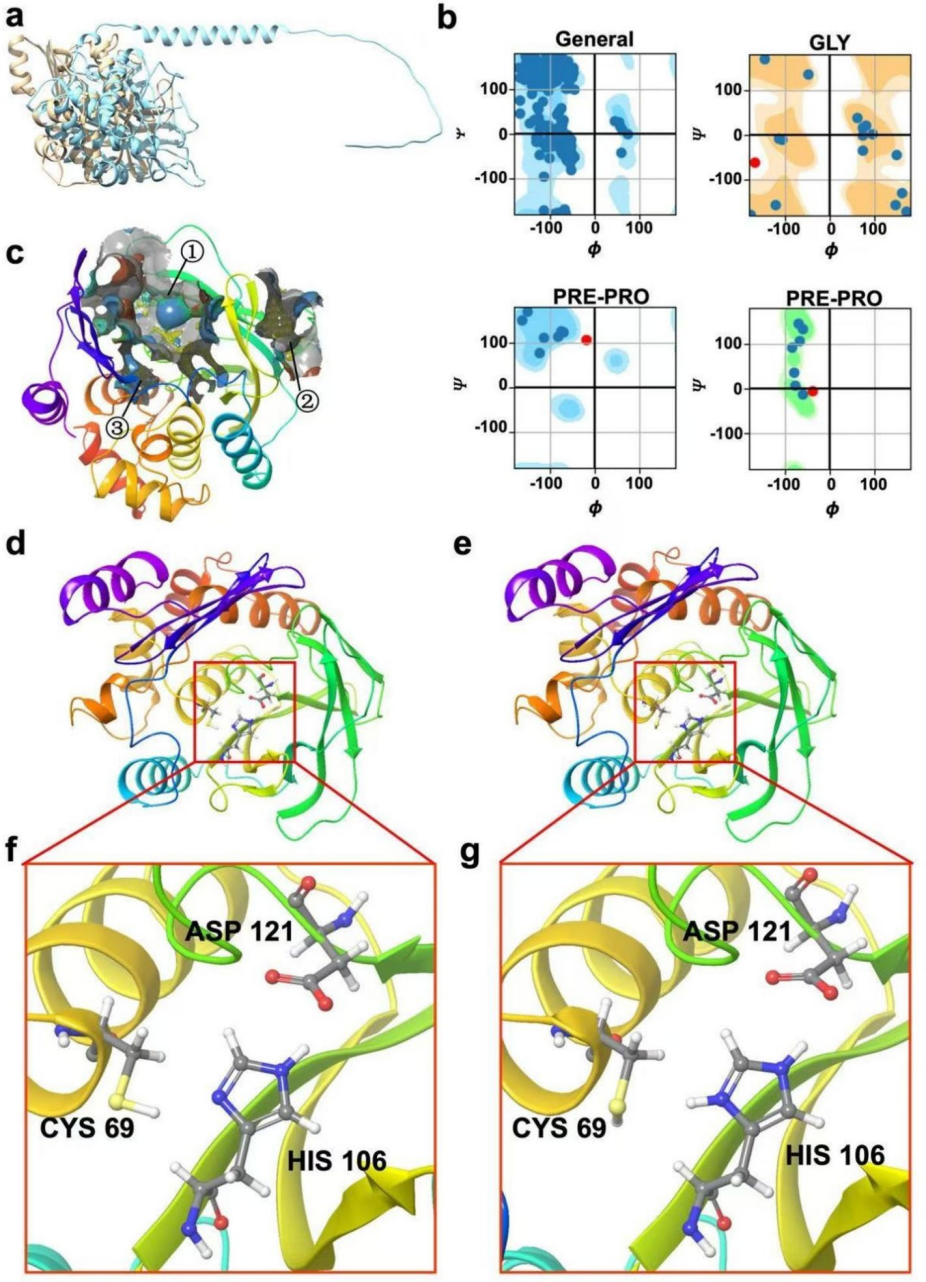
Modeling structure analysis and comparison. (**a**) Cyan is the colabfold predicted structure and brown is the crystal structure (PDBID:3LBN). (**b**) I-TASSER homology modeling structure pull-type conformational map. (**c**) Pocket predicted by SiteMap. (**d**) The I-TASSER homology modeling structure. (**e**) The SWISS-MODEL homology modeling structure. (**f**) The catalytic triplet structure of I-TASSER homology modeling structure. (**g**) The catalytic triplet structure of SWISS-MODEL homology modeling structure.

The 3D structure of the (VIBVN)NAT protein was computationally predicted using three widely accepted protein modeling software tools: I-TASSER, SWISS-MODEL, and ColabFold. These platforms are commonly employed in structural bioinformatics due to their ability to leverage different methodologies to model protein structures, enhancing the reliability of predictions by providing a comparative analysis. I-TASSER and SWISS-MODEL primarily rely on threading and homology modeling, respectively, while ColabFold, built on AlphaFold’s^11^methods, uses deep learning to predict protein structures. Considering the variability in protein folding and sequence homology, the integration of these methods allows for a more comprehensive understanding of the potential structural conformation of the (VIBVN)NAT protein. Comparison of the structure predicted by ColabFold with the homologous structure (PDB: 3LBN) of the aromatic amine n-acetyltransferase of the (VIBVN)NAT model revealed significant differences between the two structures (RMSD: 34.20 Å). One of the most prominent differences was found in the protruding loop region of the ColabFold predicted structure, which showed peculiarly substantial changes comparing with the homologous structure of (VIBVN)NAT (Fig. 2a).

Additional validation of the results was performed using Ramachandran plots In order to further assess the accuracy of the I-TASSER predictions (Fig. 2b). The Ramachandran plot is essential for evaluating the conformational viability of protein models by displaying the distribution of dihedral angles (φ and ψ) of the amino acid residues in a protein. A well-modeled protein typically exhibits the majority of its residues in favored or allowed regions on this plot^12^. The Ramachandran plot of the (VIBVN) NAT protein confirms that the predicted residue dihedral angles are located in energetically favorable regions, enhancing the accuracy of the model folding. This assessment is crucial because improper angles can result in steric clashes or strained bonds, compromising the structural integrity of the protein.

Beyond verifying the backbone geometry, we compared the predicted structure with 3LNB, a thoroughly characterized homologous structure. This comparison was instrumental in confirming the precise location of the AcCoA binding pocket (Fig. 2c). Accurate identification of the AcCoA binding site is pivotal because this pocket is directly involved in the catalytic mechanism of the protein. Correctly modeling this region is essential for any subsequent functional or biochemical studies, including inhibitor design or virtual screening for potential drug candidates. The alignment with 3LNB provided further confidence that the predicted binding pocket is correctly modeled, making it a suitable template for future studies.

The first prediction model for the structure of the (VIBVN) NAT protein by I-TASSER had a C-score of 1.84, a TM-score of 0.97 ± 0.05, and an estimated RMSD of 2.3 ± 1.7 Å (Fig. 2d). The SWISS-MODEL’s homology model for the same protein showed a GMQE score of 0.78, a QMEAN score of 0.31, and a sequence identity of 42.86% (Fig. 2e). A comparison between the homology modeling results of I-TASSER and SWISS-MODEL revealed a key difference in the protonation state of the histidine residue at position 106 (HIS 106). The protonated state of HIS 106 in the I-TASSER model appeared to be more plausible. This became particularly significant when considered in the context of the catalytic triad, a crucial region that plays an instrumental role in the enzymatic activity of (VIBVN)NAT. The accurate representation of HIS 106 is vital for maintaining the correct chemical environment at the active site and ensuring the protein’s catalytic function. This protonation state played an essential role in stabilizing the reaction intermediates during catalysis, making it a pivotal feature for the protein’s functionality (Fig. 2f and g). Therefore, the predicted structure of I-TASSER was chosen for the next stage of virtual screening.

### Druggability analysis and protein structure stability analysis

The properties of the three small molecule binding pockets predicted by SiteMap are summarized in Table S1 and illustrated in Fig. 2c. SiteMap is a widely used computational tool identifying potential binding pockets on the surface of proteins and evaluating them based on their structural and chemical properties to predict the most favorable sites for ligand interaction. Among the three identified pockets, Pocket 1 was the most suitable for small molecule binding with SiteScore and Dscore values of 1.049 and 1.084, respectively. These scores reflect key attributes of the binding pocket, such as size, enclosure, exposure to solvent, and hydrogen bond potential, which are important for the druggability of the binding pocket.

SiteScore is a metric that combines factors such as micro-pocket size, hydrophobicity, and hydrogen bonding potential, and is particularly important in assessing the suitability of micro-pockets for binding small molecules. Pocket 1 exhibited the optimal SiteScore, indicating a favorable environment for ligand binding. The Dscore, which evaluates the overall quality of the site by considering its physical and chemical properties, further confirmed that Pocket 1 is favorable for small molecule binding.

More importantly, Pocket 1 was identified as a binding site for AcCoA, an important cofactor capable of participating in a wide range of enzymatic processes including acetyl transfer. Since AcCoA binding is directly related to its enzymatic activity, identifying this binding site is essential for understanding the catalytic function of (VIBVN)NAT proteins. The favorable scores associated with Pocket 1 not only confirm its potential to bind small molecules but also emphasize its role in protein biological function. Due to the importance of this site in accommodating AcCoA, the next computational and experimental work could focus on investigating how various small molecules or inhibitors interact with this pocket to modulate protein activity.

The homology model structure generated by I-TASSER sustains a stable conformation throughout the duration of the 100 ns MD simulation process (Fig S1a, S1b).

### Water molecules prediction

The pharmacophore of the identified binding pockets was evaluated by analyzing the thermodynamic properties of water molecules within the binding pockets. Because the presence and behavior of water molecules can significantly affect ligand binding and pocket adaptation for small molecule interactions, this approach was essential. In particular, the free energy change (ΔG) associated with the displacement of water molecules from the pocket could be an important indicator of the degree of ligand binding favorability. Water molecules with a positive free energy change (ΔG > 0 kcal/mol) are energetically unfavorable and therefore more easily displaced when ligand binding occurs, which usually contributes to the stabilization of the ligand-protein complex.

**Fig. 3:**
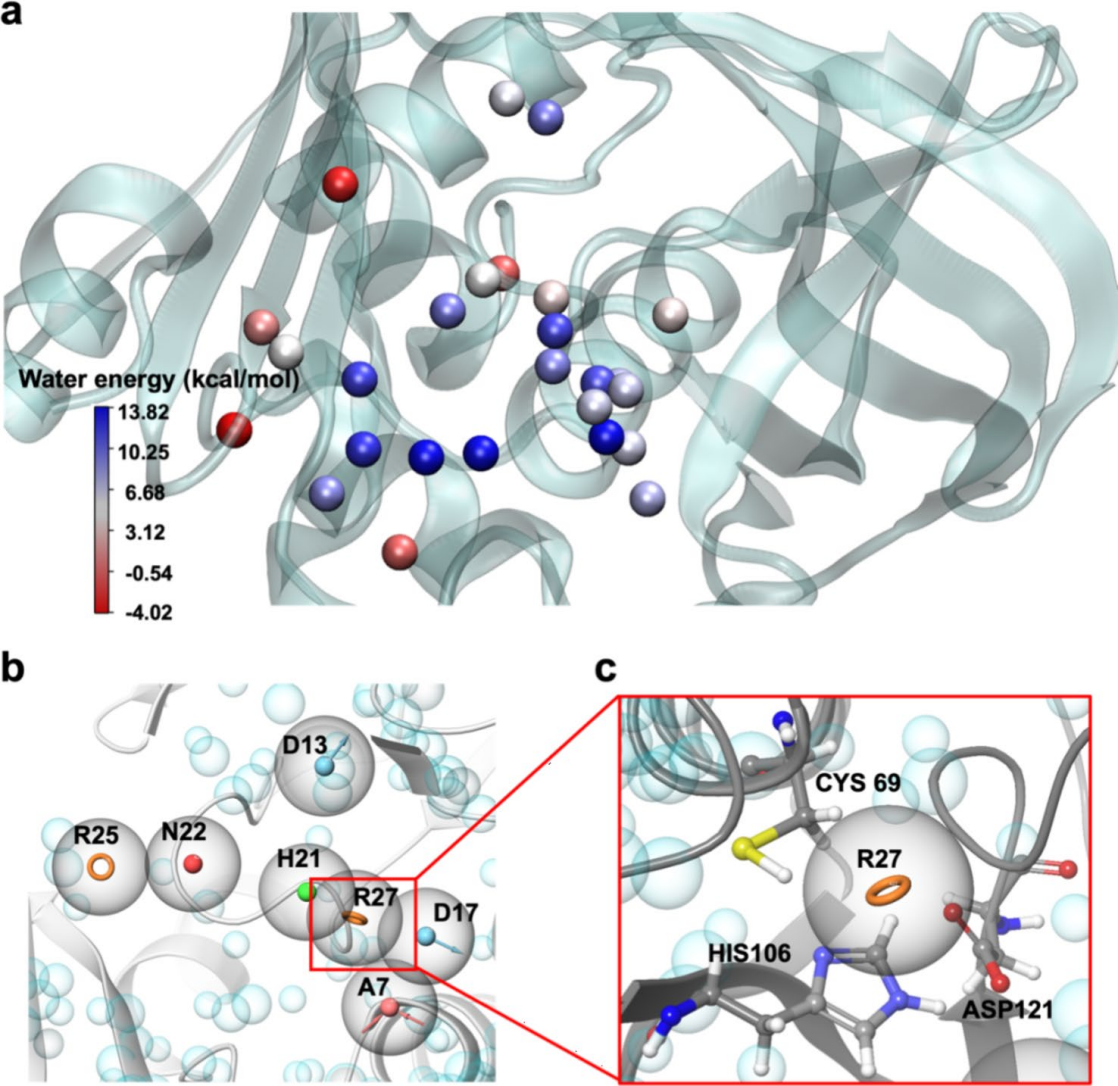
Water molecular energy and pharmacophore model. (**a**) The spherical map of water molecules is colored by the ΔG value. (**b**) The pharmacophore model based on receptor binding pocket, grey spheres represent important features pharmacophore model. R-aromatic ring, N-negatively charged group, H-hydrophobic group, D-hydrogen bond donor, A-hydrogen bond acceptor. (**c**) Catalytic triplet position generated aromatic ring pharmacophore.

Most of the water molecules in the binding pockets studied had ΔG values greater than 0 kcal/mol (Fig. 3a). This indicated that these water molecules are likely to be energetically unfavorable in their current positions and can be easily displaced by a ligand during binding. The fact that most water molecules within the pocket are replaceable by a ligand further enhances the pocket’s druggability, as the energetic cost of displacing these water molecules is compensated by the favorable interactions that arise when a ligand occupies their positions. On the other hand, a minority of water molecules display ΔG values far less than 0 kcal/mol, suggesting that they are energetically favorable in the pocket and may be more challenging to displace. However, their presence does not significantly impact the overall druggability of the pocket, given that the majority of water molecules are replaceable.

The replaceability of water molecules is a key factor in determining the suitability of a binding pocket for ligand binding. Water molecules that can be displaced and replaced by a ligand contribute to the overall stabilization of the ligand-protein complex. In this case, the thermodynamic properties of the water molecules suggested that the binding pocket is highly favorable for ligand interactions. Once the ligand occupies the position previously occupied by the water molecule, the complex is stabilized, further supporting the potential of the pocket as a druggable site.

### Pharmacophore model

The pharmacophore models consisting of seven features named as RRNHDDA (R-aromatic ring, N-negatively charged group, H-hydrophobic group, D-hydrogen bond donor, A-hydrogen bond acceptor) were successfully generated (Fig. 3b). The pharmacophore model was constructed based on the receptor, identifying key pharmacophore features from critical interactions between (VIBVN) NAT and the substrate AcCoA. This process resulted in the RRNHDDA pharmacophore. A total of 20,000 compounds were selected from the Specs database based on their compatibility with the RRNHDDA pharmacophore model. Selection was determined by ranking compounds according to their alignment with the pharmacophore features, including both positional and physicochemical property matches. These top-ranked compounds were subsequently subjected to molecular docking for further evaluation.

### Molecular docking, MM/GBSA and strain energy

The top 200 ligands were selected according to their watvina docking scores. 138 small molecules exhibiting ΔG_*MM/GBSA*_ < -40 kcal/mol were chosen. Subsequently, 113 small molecules with ligand strain energies < 10 kcal/mol were retained (Table 1).

**Table 1:** Information about 20 ligands.

ID	Docking_Score (kcal/mol)	MM/GBSA (kcal/mol)	Strain energy (kcal/mol)	Aromatic carbons (number)
AF-399-13806047	− 8.6	− 59.81	6.72	3.00
AF-399-13909156	− 8.2	− 48.58	2.30	2.00
AF-399-42679396	− 8.0	− 45.05	4.55	2.00
AG-205-36710025	− 8.0	− 43.42	2.05	3.00
AG-205-36953353	− 9.3	− 57.06	5.54	1.00
AG-670-34811031	− 8.2	− 45.70	0.97	2.00
AH-487-15274472	− 8.3	− 57.80	6.17	2.00
AH-487-40687107	− 8.5	− 50.09	6.97	2.00
AH-487-41186996	− 8.9	− 45.80	1.83	3.00
AK-968-11563024	− 8.1	− 47.85	4.96	1.00
AK-968-40605601	− 8.2	− 45.08	8.92	2.00
AK-968-41017295	− 8.3	− 56.93	5.68	1.00
AN-153-12399121	− 8.8	− 53.53	9.24	2.00
AN-329-15332092	− 8.1	− 62.69	6.38	1.00
AN-329-41044423	− 8.1	− 42.21	6.46	2.00
AN-329-42685695	− 8.2	− 54.84	1.41	3.00
AN-648-14354004	− 8.3	− 47.68	6.05	3.00
AN-919-15529089	− 8.5	− 54.15	1.75	1.00
AN-988-40680384	− 8.1	− 51.87	8.65	1.00
AO-299-15047104	− 8.1	− 56.64	3.62	1.00

### Screening for specific pharmacophore characteristics

Watvina was used to identify pharmacophores that interact with the catalytic triad, a key element of (VIBVN)NAT’s catalytic activity. Ligands containing aromatic carbons at binding site were preferred (Fig. 3c). In summary, 20 ligands with aromatic carbon at this position were identified (Table 1) and procured for further test.

### Screening of inhibitors

**Fig. 4:**
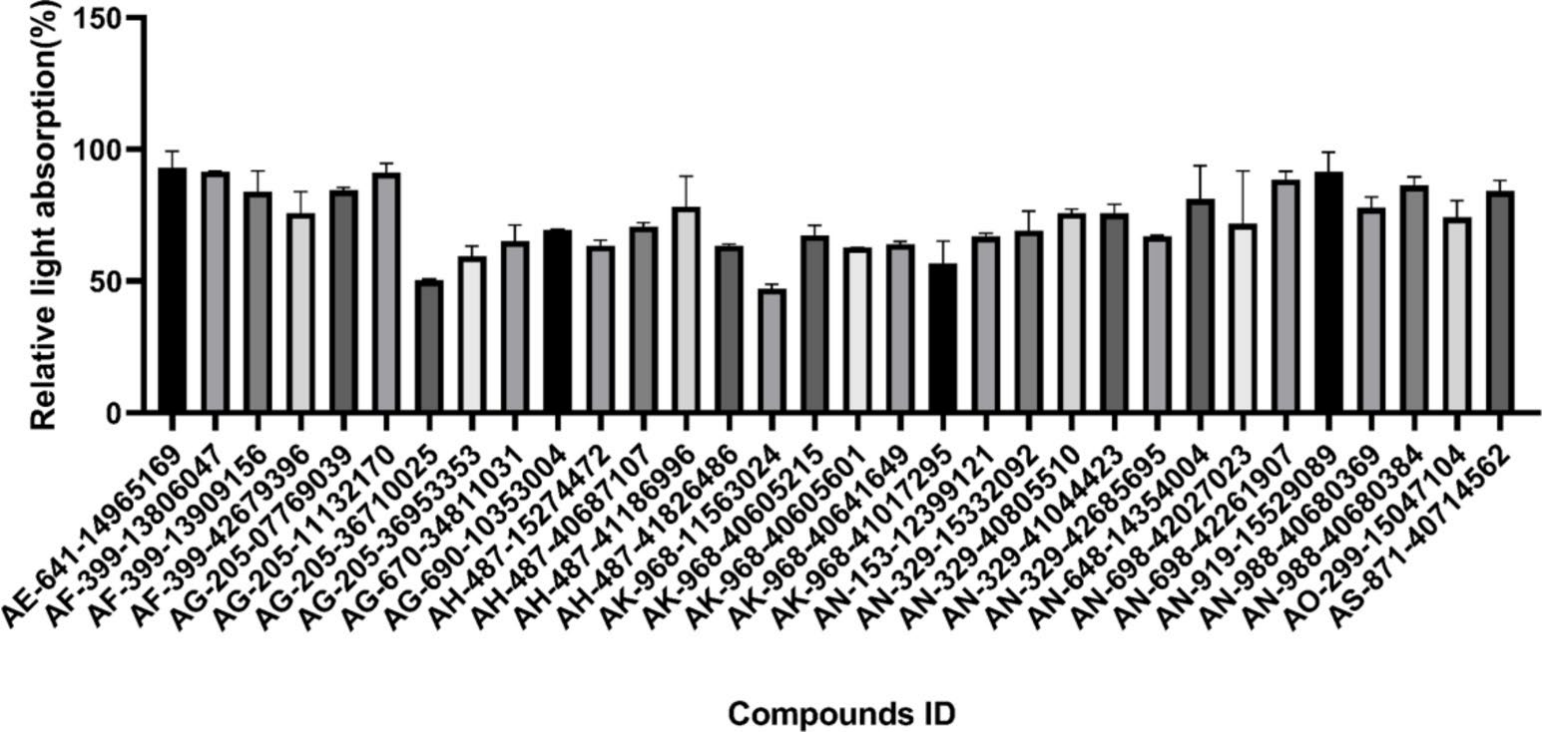
The relative absorbance of the compound at final concentration of 10 µM.

**Fig. 5:**
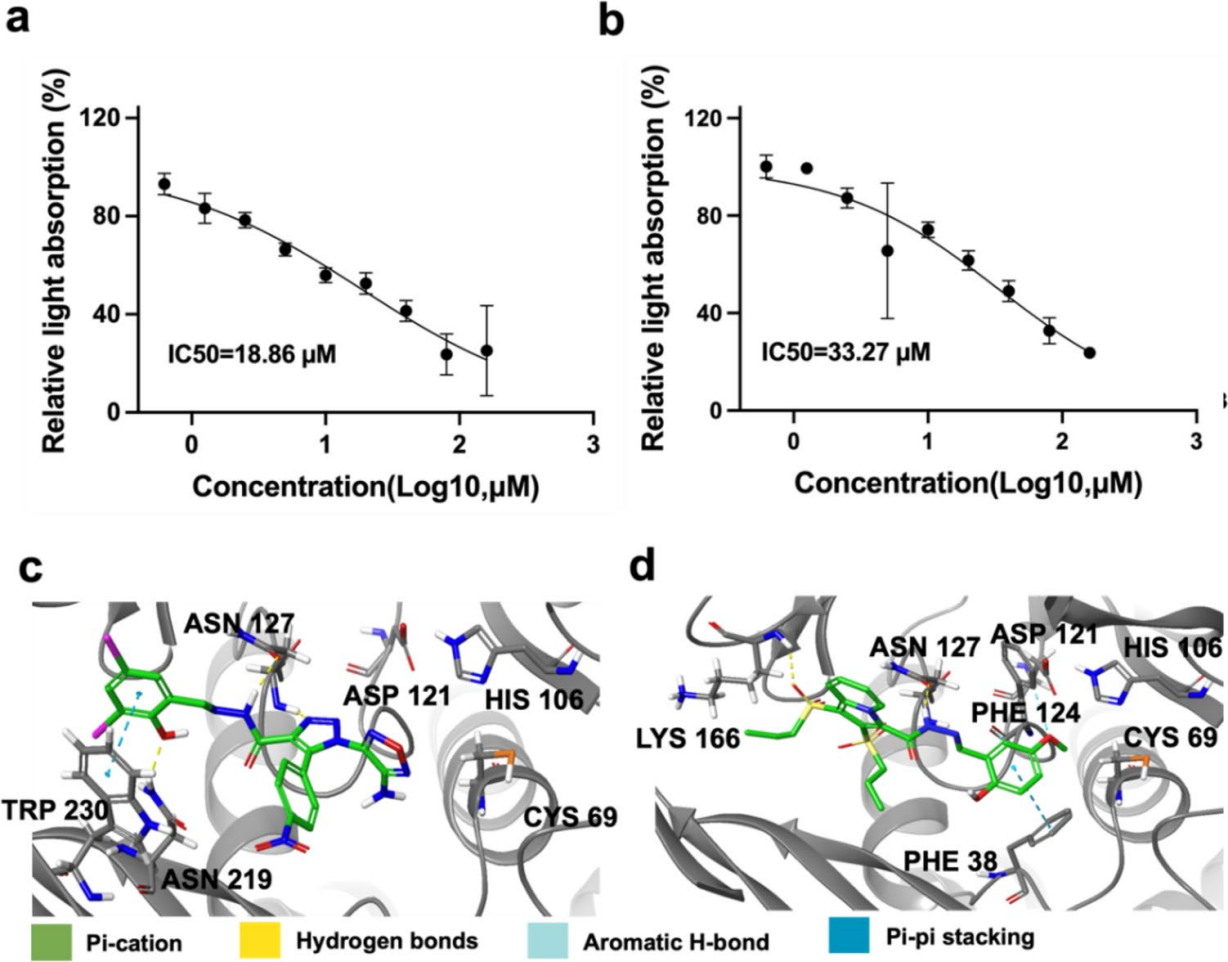
Structure activity relationship analysis of AK-968-11563024 and AG-205-36710025. The relative absorbance of AK-968-11563024 (**a**) and AG-205-36710025 (**b**) were measured. (**c**) The interaction of AK-968-11563024 and (VIBVN)NAT. (**d**) The interaction between AG-205-36710025 and (VIBVN)NAT.

The concentration of 10 µM was initially used for preliminary screening of compounds, selecting those with notable relative inhibitory activity (Fig. 4). Whereafter, we identified two novel (VIBVN)NAT inhibitors, AK-968-11563024 (IC_50,_ 18.86 µM) and AG-205-36710025 (IC_50,_ 33.27 µM) (Fig. 5a and b).

The molecular docking results between (VIBVN)NAT and AK-968-11563024 were analyzed. The results revealed that the best inhibitory compound AK-968-11563024 was found to form an interaction with LYS168 TRP230 PHE124 ASN127 HIS167 LYS166 of (VIBVN)NAT (Fig. 5c). However, the compound AG-205-36710025 was found to interact with GLN97 ASN96 PHE205 amino acid of (VIBVN)NAT (Fig. 5d).

### Molecular dynamics analysis

**Fig. 6:**
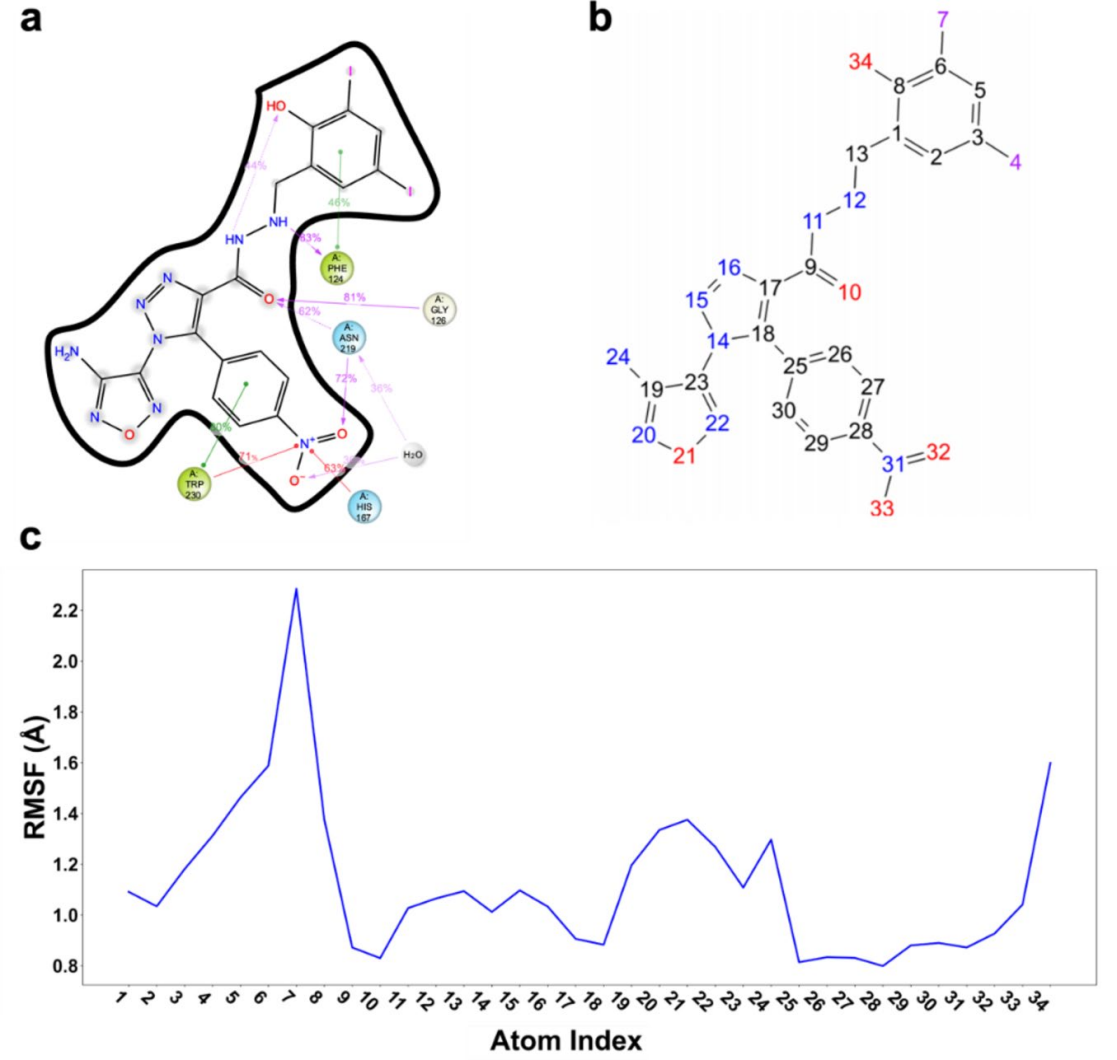
AK-968-11563024 MD analysis. (**a**) 2D interaction of (VIBVN)NAT and AK-968-11563024 during MD simulation. (**b**) Atomic serial number corresponding to the RMSF of AK-968-11563024. (**c**) L-RMSF of the ligand during MD simulation.

During the 100 ns MD simulations, the RMSD of the (VIBVN)NAT in AK-968-11563024 fluctuates by less than 2.7 Å, which is considered to have reached a equilibration state in the simulations (Fig S2a). The SSE of the (VIBVN)NAT in AK-968-11563024 around 50%, indicating that the protein has stable secondary structure (Fig S2b). We focused on the frequent interactions (more than 30%) of AK-968-11563024 and the results showed a large number of hydrogen bonds between AK-968-11563024 and amino acids, especially at the binding site. This demonstrated AK-968-11563024 mainly interacts with PHE124, GLY126 and ASN219 (Fig. 6a). All interactions were counted and it was found that ASN219 produced the most interactions (Fig S2c). This underscored AK-968-11563024’s potential as a potent (VIBVN)NAT inhibitor.

AK-968-11563024 was also found to be able to form hydrophobic interactions with PHE124 and TRP230, and cation-π interactions with HIS167 and TRP230. Additionally, the presence of π-π superposition interactions between AK-968-11563024 and TRP230 and PHE124 was similarly observed in our study. To better understand the structure-activity relationships, we evaluated the stability of the ligands within the complexes. The ligand root-mean-square fluctuation (L-RMSF) analysis showed that the internal atomic fluctuations of the ligands were relatively stable, as depicted in Fig. 6b and c. Overall, the complex between AK-968-11563024 and (VIBVN)NAT demonstrated stability throughout the duration of the MD simulations.

We also performed simulations for AG-205-36710025 and found that the molecule stably binds to the binding pocket (Fig S3a), forming significant interactions with LYS166, TRP230, GLY126, and ASN127 (Fig S3b). Notably, TRP230 and GLY126 are the same amino acids that interact significantly with AK-968-11563024, suggesting that TRP230 and GLY126 may play a crucial role in the activity.

## Discussion

In this study, we proposed a computational strategy with the goal of identifying potential inhibitors for the (VIBVN) NAT protein. This method involves predicting the druggability of binding pockets, which is based on the thermodynamic properties of water molecules. The principal emphasis of this methodology is on the execution of molecular docking via watvina, coupled with meticulous screening of specific pharmacophores. Initially, 200,000 molecules from the Specs database were subjected to pharmacophore-based screening. These molecules were then docked using watvina, yielding the top 200 performing candidates. A comprehensive evaluation was then performed using MM/GBSA and strain energy calculations. Subsequent pharmacophore characterization via watvina identified two compounds, AK-968-11563024 and AG-205-36710025 with IC50 values of 18.86 µM and 33.27 µM, respectively. This observation implies that AK-968-11563024 exhibits a more potent inhibitory impact on the (VIBVN) NAT protein than does AG-205-36710025. MD analysis of the AK-968-11563024 complex revealed its structure-activity relationship. AK-968-11563024 forms numerous hydrogen bonds with PHE124, GLY126, and ASN219, hydrophobic interactions with PHE124 and TRP230, and cationic-π interactions with HIS167 and TRP230. It’s also involved in π-π stacking with TRP230 and PHE124. These interactions stabilize the complex, indicating AK-968-11563024’s strong binding to the (VIBVN) NAT protein.

In conclusion, we have identified potential (VIBVN)NAT inhibitors through an extensive computational approach and offered valuable insight into the structure-activity relationship. This work contributes to the understanding of (VIBVN) NAT inhibition and paves the way for future drug design and development. This may provide a framework for the discovery and design of potential drug candidates, contributing to the advancement of targeted therapeutics.

## Conclusions

**Fig. 7:**
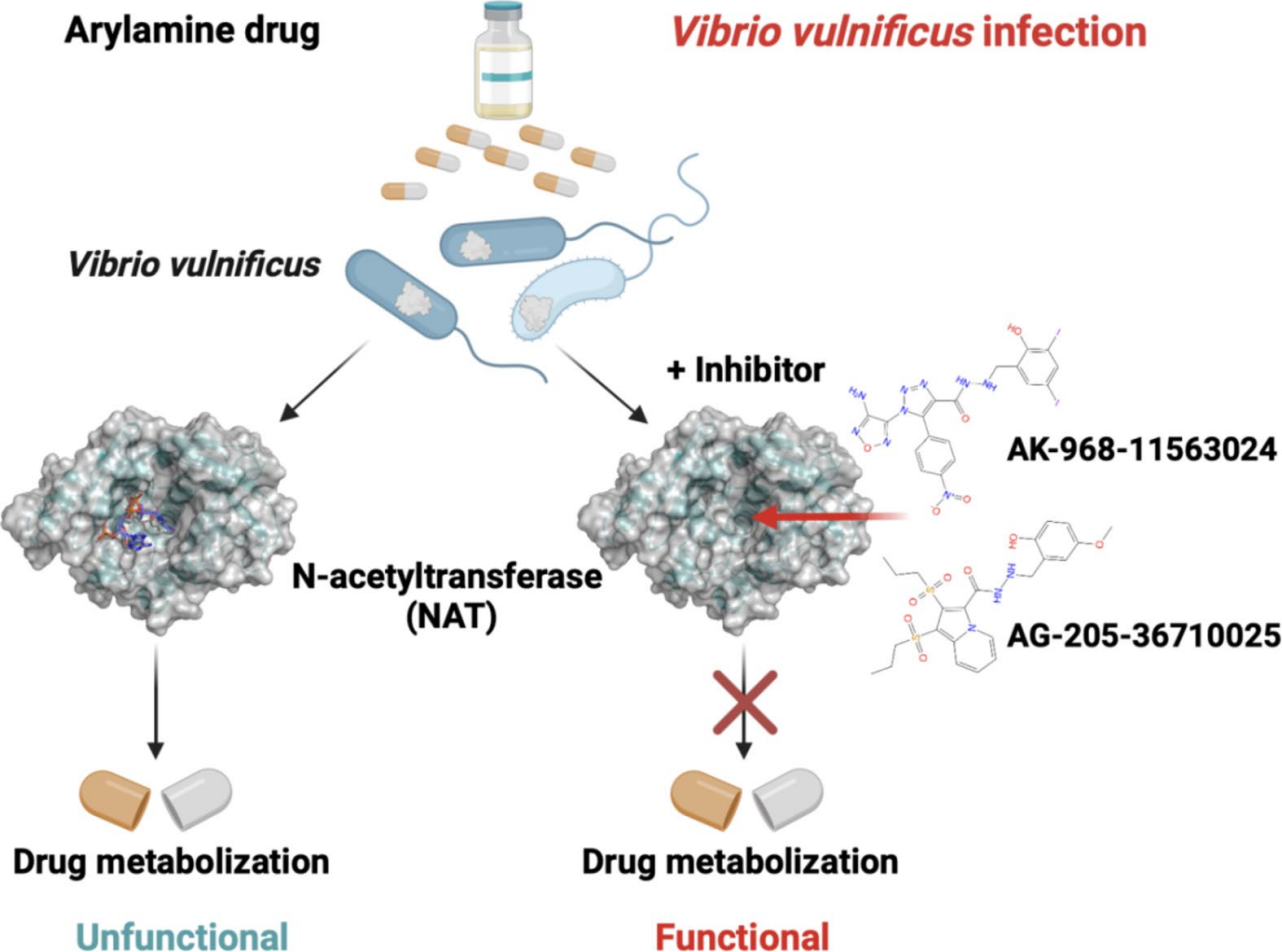
Schematic representation of AK-968-11563024 and AG-205-36710025 preventing NAT from metabolizing aromatic drugs by occupying the AcCoA binding site.

The novel NAT inhibitors of marine *V. vulnificus*, namely AK-968-11563024 and AG-205-36710025 were primarily identified through virtual screening to suppress NAT from metabolizing aromatic drugs by occupying the binding site of AcCoA (Fig. 7). Therefore, these two molecules could serve as potential therapeutic candidates for preventing resistance in marine *V. vulnificus.*

## Materials and methods

### Homologous modeling and ColabFold

I-TASSER^13,14^ (https://zhanglab.dcmb.med.umich.edu/I-TASSER/), SWISS-MODEL^15,16^ (https://swissmodel.expasy.org/) and ColabFold^17^ were used to predict the structure of (VIBVN)NAT (UniProt id: A0A4V3BB88). The structure predicted by ColabFold is superimposed on the homologous structure associated with Protein Data Bank^18^ (PDB) ID:3LBN^19^. The resulting structures were then comparatively analyzed using a pull-type conformational map (https://github.com/gerdos/PyRAMA^12^).

### Protein preparation

The predicted structure of (VIBVN)NAT protein was prepared using the Protein Preparation Wizard^20^ module [Schrödinger Release 2020-2, Protein Preparation Wizard, Schrödinger, LLC, New York, NY, 2020.] to add hydrogen. The hydrogen bonding network was optimized using PROPKA^21,22^ with a value of PH 7.0. Subsequently, the structure was minimized using the OPLS3e^23^ force field until the RMSD value of the heavy atoms converged to 0.3 Å. The binding site of AcCoA is also the binding site of the competitive small molecule inhibitor.

### Druggability analysis

The binding pocket in (VIBVN)NAT was analyzed by submitting the prepared protein structure to the Schrödinger’s SiteMap^24,25^ module [Schrödinger Release 2020-2, Sitemap, Schrödinger, LLC, New York, NY, 2020]. MD simulations of (VIBVN)NAT apo structure were performed using the Desmond^26^ module [Schrödinger Release 2020-2, Desmond, Schrödinger, LLC, New York, NY, 2020] to determine the structural stability of the proteins. All simulations were performed using the OPLS3e force field. The simulation system was assembled using the simple point charge (SPC) water molecular model to create a cubic water box. To achieve electroneutrality, Na^+^ and Cl^−^ were incorporated into the system. Further, a buffer solution with a salt ion concentration of 150 mM was introduced to mirror the experimental conditions. A harmonic position constraint of 5 kcal/mol/Å^2^ was enforced on the protein’s main chain atoms. Subsequently, the system was subjected to an energy minimization process for a duration of 100 ps. This step enabled sufficient equilibration between the protein and water molecules, effectively eliminating any potential collisions between the amino acid side chains of the protein and hydrogen atoms. In the equilibrium system under NVT, 2.5 kcal/mol/Å^2^ positional constraints were applied to protein backbone atoms at 300 K and 2 ns MD simulations were performed to fully equilibrate the water molecules. The procedure used NPT, 2.5 kcal/mol/Å2 to reconcile the equilibrium system under positional constraints, and 5 ns thermostatic calorimetry simulations of protein backbone atoms at 300 K and 1 atm to fully relax the protein amino acid side chains. The final phase involved a 100 ns MD simulation, which was executed using a Nose-Hoover chain thermostat and an Andersen-Hoover barometer at conditions of 300 K and 1 atm. The simulation was performed with a 2 fs timestep, and a cutoff radius of 9.0 Å was set for computing long-range interactions. MD simulation trajectories were generated at intervals of 20 ps, resulting in a total of 5000 trajectories.

### Water molecule prediction

AmberTools20^27^’s (https://ambermd.org/AmberTools.php) tleap was used to process the protein structure. The protein was parameterized using the ff19SB force field^28^, and a water cubic box was created using the optimal point charge (OPC) force field^29^. Na^+^ and Cl^−^ were added to maintain electrical neutrality in the system. Subsequently, restricted MD simulations of the apo structure were conducted using Amber20. The system was minimized under periodic boundary conditions, with constraints applied to the protein’s heavy atoms and a simple harmonic constraint imposed with a force constant of 10 kcal/mol/Å^2^. The minimization process comprises 20,000 steps, utilizing the gradient descent algorithm for the first 2,500 steps and the conjugate gradient algorithm for the remaining 17,500 steps. A cutoff radius of 10.0 Å is set for these operations. Thus, the minimization is primarily executed using the gradient descent algorithm. Following, the system is equilibrated and the temperature is set to 300 K. The constraints on the protein’s heavy atoms are gradually removed at NVT, reducing to a force constant of 2.5 kcal/mol/Å^2^, and the system is allowed to equilibrate for 125 ps. During the 2 ns NPT pre-equilibrium phase, we established conditions with a temperature of 300 K, a pressure of 1 atm, and a force constant of 2.5 kcal/mol/Å^2^ for protein’s heavy atoms. Lastly, restricted MD simulations were carried out for 30 ns in the NPT system within temperature of 300 K, the pressure of 1 atm, and the protein’s heavy atoms constant of 2.5 kcal/mol/Å^2^. Temperature regulation was achieved using the SHAKE algorithm, which was confined to the hydrogen atoms via a Langevin thermostat with a collision frequency of 1 ps^− 1^. The pressure was controlled using the Berendsen pressure regulator with an isotropic scale and a coupling constant of 1 ps. A time step of 2 fs was set and data was saved every 1 ps, resulting in a trajectory file containing 30,000 frames.

The water molecules were studied using the GIST^30^ method. Moreover, we analyzed the water molecules data using DBSCAN^31^ algorithm. This helped us understand the positions and energies of water molecules in the binding pocket.

### Ligands preparation

As a virtual screening library, Specs database (www.specs.net) contains over 210,000 small drug-like compounds. To make sure these compounds had the right shape during screening, the Ligprep module [Schrödinger Release 2020-2, Ligprep, Schrödinger, LLC, New York, NY, 2020.] was employed for conformation generation and preparation. The Epik^32^ program was utilized to generate potential ionized states within a pH range of 7.0 ± 2.0. This process involved transforming the 2D structure of the small molecule into a 3D conformation. Subsequent energy minimization was performed to optimize these conformations.

### Pharmacophore hypothesis generation and database screening

The Phase^33,34^ module [Schrödinger Release 2020-2, Phase, Schrödinger, LLC, New York, NY, 2020.] was utilized to create pharmacophore models. We selected the top 20,000 matches for further molecular docking after screening the Specs database using these models.

### Molecular docking

Watvina^35^ (https://github.com/biocheming/watvina, accessed on 30 November 2021, Ximing Xu, Qingdao, China) was used for molecular docking and screening specific pharmacophore profiles. Both ligands and predicted protein structures were converted to PDBQT format using rdkit2pdbqt.py^36^ (https://github.com/biocheming/watvina, accessed on 30 November 2021, Ximing Xu, Qingdao, China). AcCoA helped us define docking box. With watvina’s default settings, we docked molecules and selected the top 200 small molecules.

### MM/GBSA and ligand strain energy

The Prime^37,38^ MM/GBSA module [Schrödinger Release 2020-2, Prime, Schrödinger, LLC, New York, NY, 2020.] was used to calculate the binding free energy between 200 small molecules and (VIBVN)NAT proteins.

### Screening based on specific pharmacophore characteristics

An examination of the 3D structure of the (VIBVN)NAT protein showed the vital role of the centrally positioned aromatic ring pharmacophore feature within the catalytic triad in the catalytic process. As a result, we used watvina to screen ligands that have aromatic carbon atoms at this position and ranked them based on the number of such atoms. We selected and purchased 20 ligands with aromatic carbons for subsequent experimental validation.

### (VIBVN)NAT enzyme activity detection and inhibitor screening

The (VIBVN)NAT enzyme activity was assayed based on previous study^10^ via DTNB method. 5,5-dithio-(2-nitrobenzoic acid) (DTNB reagent) was employed to determine AcCoA-dependent acetylation in a reaction system of 100 µL. The reaction was stopped by the addition of 50 µL cold DTNB in 6 M guanidine chloride (GdmCl) (2.0 mg/mL, final concentration) to stop the reaction. The enzyme activity was measured as the change in absorbance at 405 nm.

### Molecular dynamics simulation

MD simulations were conducted using the Desmond module [Schrödinger Release 2020-2, Desmond, Schrödinger, LLC, New York, NY, 2020.] with all simulations operating within the OPLS3e force field. We optimized and minimized the Holo structure using the Protein Preparation Wizard module, employing the identical approach as in the MD simulations of the Apo structure.

## Electronic supplementary material

Below is the link to the electronic supplementary material.

Supplementary Material 1

## Data Availability

Data is provided within the manuscript or supplementary information files; further inquiries can be directed to the author, Ximing Xu: xuximing@ouc.edu.cn.
