# Supplementary material for: Novel inhibitors of the (VIBVN) NAT protein identified through pharmacophore modeling
Source: Sci Rep. 2025 Jan 23;15:2898. doi: 10.1038/s41598-025-85869-4 (PMC11754874; doi:10.1038/s41598-025-85869-4)
Supplement: Supplementary file 1 — Supplementary Material 1 [file 41598_2025_85869_MOESM1_ESM.pdf]

Novel inhibitors of the (VIBVN) NAT protein identified through pharmacophore modeling

Wei Wei[1, 3, #], Xionghao Li[2, 3, 4, #], Ning Hou[2], Aowei Xie[5], Huicong Liang[2], Ting Gao[5], Xiaoli Jing[4], Liqin Li[1, 6, *], Jiejie Hao[2, *], Ximing Xu[2, 3, 4, *]

1. Affiliated Huzhou Hospital, Zhejiang University School of Medicine; The Key Laboratory of Molecular Medicine, Huzhou Central Hospital; The Fifth School of Clinical Medicine of Zhejiang Chinese Medical University, Huzhou 313000, China.

2. Key Laboratory of Marine Drugs, Ministry of Education, School of Medicine and Pharmacy, Ocean University of China, Qingdao 266071, China.

3. Marine Biomedical Research Institute of Qingdao, Qingdao 266071, China.

4. Network and Information Center, Qingdao Marine Science and Technology Center, Qingdao 266237, China.

5. College of Food Science and Engineering, Ocean University of China, Qingdao 266071, China.

6. TCM Key Laboratory Cultivation Base of Zhe jiang Province for the Development and Clinical Transformation of Immunomodulatory drugs, Huzhou Central Hospital, Huzhou 313000, China.

*Authors for correspondence: Ximing Xu, xuximing@ouc.edu.cn, Liqin Li, liliqin@hzhospital.com, Jiejie Hao, 2009haojie@ouc.edu.cn.

[#]Wei Wei and Xionghao Li contributed equally to this work.

The authors have declared that no conflicts of interests exist.

Supplementary Materials

Site ID	SiteScore	Size	Dscore	Volume	Exposure	Enclosule	Contact	Phobic	Philic	Balance	Don/acc
**1**	1.049	110	1.084	350.76	0.651	0.733	0.808	0.782	0.907	0.862	0.652
**2**	0.919	43	0.969	80.734	0.427	0.73	1.033	4.077	0.261	15.632	0.497
**3**	0.79	21	0.642	44.118	0.447	0.978	1.635	2.57	1.153	2.23	4.48

Table S1 Pocket informations predicted by SiteMap.

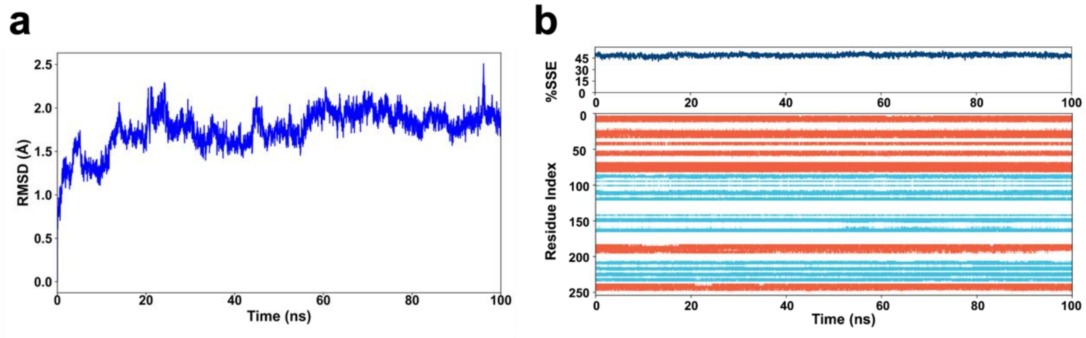

Figure S1. Apo structure MD analysis. (**a**) RMSD of the Apo structure during 100 ns MD simulation. (**b**) Protein SSE change during 100 ns MD simulation.

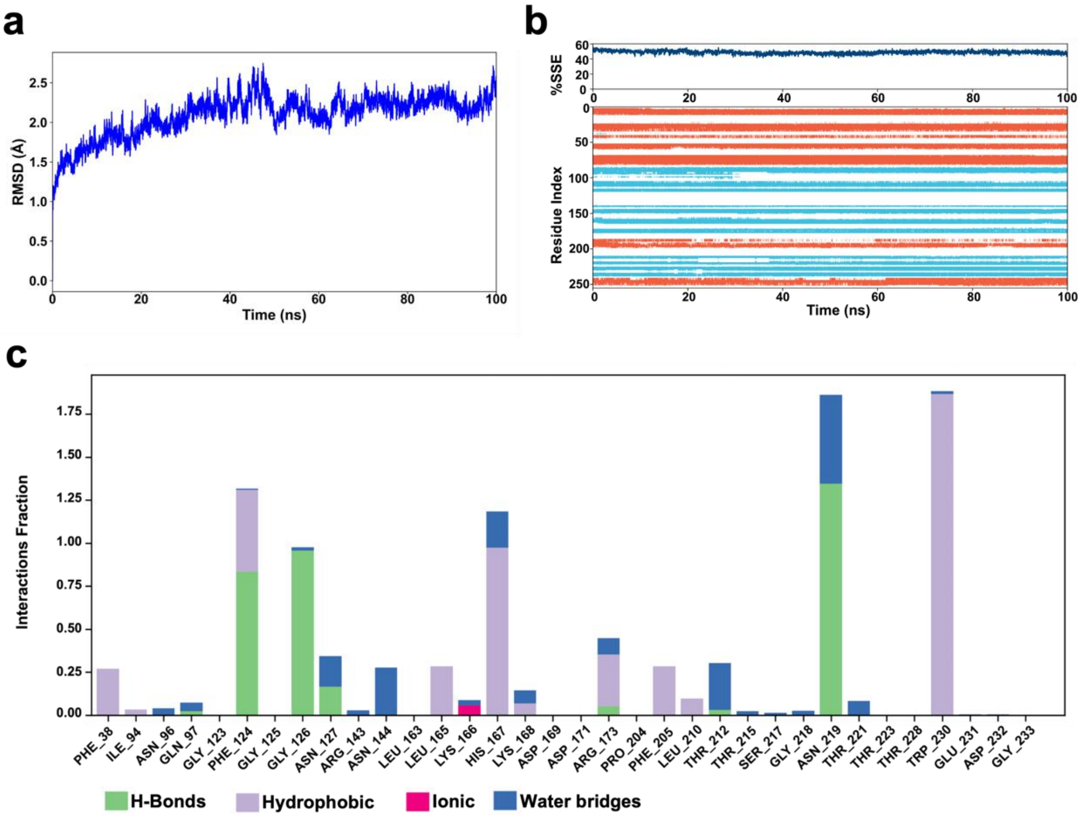

Figure S2. AK-968-11563024 MD analysis. (**a**) RMSD of the Holo structure during 100 ns MD simulation. (**b**) Protein SSE change during 100 ns MD simulation. (**c**) The main interactions between (VIBVN)NAT and AK-968-11563024 during 100 ns MD simulations.

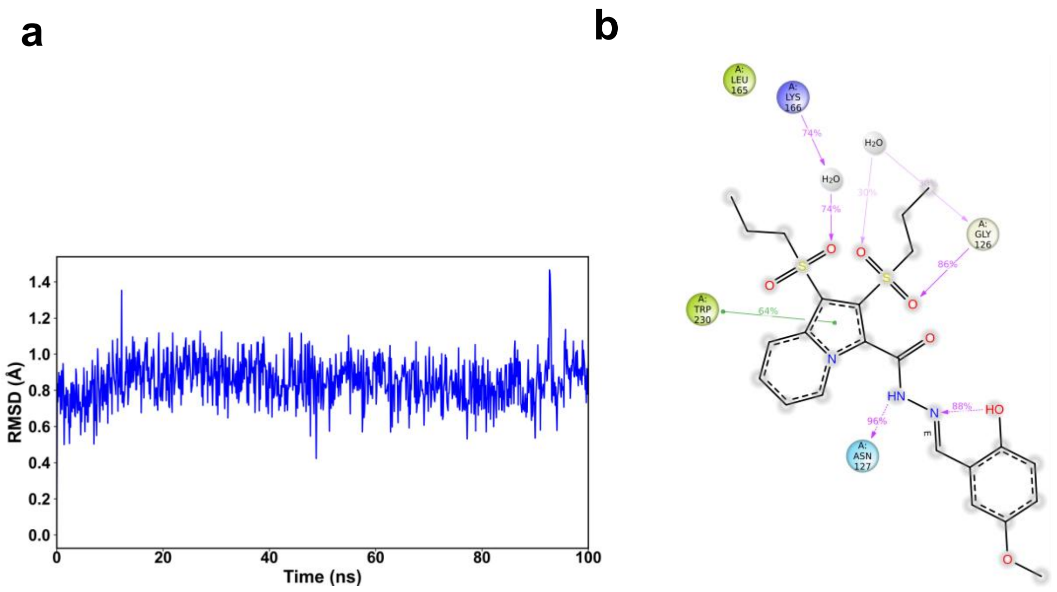

Figure S3. AG-205-36710025 MD analysis. (**a**) RMSD of the Holo structure during 100 ns MD simulation. (**b**) 2D interaction of (VIBVN)NAT and AG-205-36710025 during MD simulation.
